# PAIN, AFFECT, AND OBJECTIVE ACTIVITY LEVELS IN OLDER ADULTS WITH OSTEOARTHRITIS

**DOI:** 10.1093/geroni/igz038.2633

**Published:** 2019-11-08

**Authors:** Emily A Behrens, Jason A DeCaro, Dylan M Smith, Patricia A Parmelee

**Affiliations:** 1 The University of Alabama, Tuscaloosa, Alabama, United States; 2 Stony Brook University, Stony Brook, New York, United States

## Abstract

This research examined the relation between physical activity, pain, and mood among older adults with osteoarthritis (OA). Physical activity is associated with long-term maintenance of function in persons with chronic pain (Dunlop et al., 2014), but less is known about the association between objective measures of activity and transient mood states. Therefore, we captured the activity and mood levels of 218 older adults with knee OA over a seven-day period. Wrist and waist accelerometers captured small and large motor movements. Self-reported momentary pain and affect were collected through phone calls four times daily. We examined average and peak activity levels over the 4-hour windows between self-reports. Cross-sectionally, there was no association between momentary pain and activity. Average large motor movement was positively associated with positive affect and negatively associated with negative affect. Analyses revealed one association between affect and average previous activity; small motor movements predicted greater positive affect. Peak levels of both movements predicted greater positive affect, but only peak wrist activity predicted negative affect. Peak small motor movement at the previous call was associated with both positive and negative affect. These results provide insight into the unique contributions of small and large motor activity to mood and pain states. It appears that average large motor movements and prior small motor activity may have the greatest impact on momentary affect. Further study of distinct activity types and mood will be important for understanding and improving the quality of life among individuals diagnosed with OA (Supported by R01-AG041655).

